# A Saudi Girl With Co-occurring CHD1 (Pilarowski-Bjornsson Syndrome) and ASH1L Gene Variants

**DOI:** 10.7759/cureus.49905

**Published:** 2023-12-04

**Authors:** Maryam Al-Aamri, Moayad Alshaqaq, Sameer Y Al-Abdi

**Affiliations:** 1 Pediatric Nephrology, Maternity and Children Hospital Alahsa, Alahsa, SAU; 2 Pediatric Neurology, Maternity and Children Hospital Alahsa, Alhasa, SAU; 3 Pediatrics/Neonatology, Al Ahsa Hospital, Alahsa, SAU

**Keywords:** autism, hypotonia, neurodevelopmental disorder, chd1, chromodomain helicase dna-binding-1, chromatin remodeler, pilarowski-bjornsson syndrome

## Abstract

Pilarowski-Bjornsson Syndrome (PBS) is a recently identified and rare genetic disorder. PBS is caused by missense variants in the CHD1 gene, a chromatin remodeler and helicase DNA-binding protein. In this report, we present the first case of PBS in Saudi Arabia. The patient exhibits a phenotype and genotype that are consistent with previously reported cases of PBS. Notably, this case is unique due to the coexisting presence of an absent, small, and homeotic disks protein 1 homolog like a histone lysine methyltransferase (ASH1L) variant and developmental dissociation. The ASH1L variant may contribute to the developmental dissociation observed in the patient. Furthermore, since the patient is female, this case contributes to the female-skewed distribution of PBS, although the exact cause of this phenomenon requires further investigation. This report highlights the importance of identifying and characterizing rare genetic disorders such as PBS. Understanding the genetic basis of these disorders can lead to improved diagnosis, treatment, and management strategies. Continued research on the genetic and molecular mechanisms underlying PBS and related disorders is crucial for advancing our knowledge and developing effective therapies.

## Introduction

Pilarowski-Bjornsson syndrome (PBS) is a rare Mendelian disorder of the epigenetic machinery caused by missense variants in the chromatin remodeler chromodomain helicase DNA-binding-1 (CHD1) gene [1]. It was first reported in 2018, and only six cases have been reported worldwide to date [1,2]. However, the precise prevalence of PBS is yet to be determined, as it has recently been discovered. With time, the real prevalence of the condition will become more known. PBS is mainly characterized by hypotonia, global developmental delay, autism, speech apraxia, seizures, growth retardation, and craniofacial dysmorphism, and it predominantly affects females [1,2]. Five of the six reported cases are female [1,2]. The underlying reason for the higher prevalence of female cases in this context remains unclear [1,2]. However, it has been hypothesized that male individuals may exhibit a lower tolerance to CHD1 variants [1].

The CHD1 gene is widely expressed across various tissues, including the brain, bone marrow, lymph nodes, intestines, ovary, and testis [1,2]. However, the highest expression levels are observed in the cerebellum and basal ganglia [1]. Recent studies have implicated the cerebellum in the manifestation of autistic spectrum disorder (ASD) [1]. CHD1 may play a critical role in the development of cranial neural crest and jaw cartilage formation, which are essential for craniofacial development [2].

The gene encoding the absent small and homeotic disks protein 1 homolog like histone lysine methyltransferase (ASH1L) is expressed at high levels in the prefrontal cortex, a key brain region responsible for cognitive, emotional, and social function [3,4]. As a result, ASH1L is considered a primary risk factor for autism spectrum disorder (ASD) and intellectual disability [3,4]. However, the biological mechanisms underlying these associations remain poorly understood [3,4].

We are reporting the first case from Saudi Arabia that is consistent with the phenotype and genotype of PBS. A unique aspect of this case is that it shows developmental dissociation and both CHD1 and ASH1L variants coexist. The present report aims to enhance awareness among healthcare providers in Saudi Arabia, thereby promoting improved diagnosis and management of PBS. Furthermore, the report is expected to contribute to the global understanding of this syndrome.

## Case presentation

A female patient was born to Saudi parents at 36 weeks of gestation and weighed 2.08 kilograms, which was just on the 10th percentile of the Fenton growth chart [5]. She was delivered via emergency cesarean section due to decreased fetal movement, suspected fetal compromise, and meconium-stained amniotic fluid. She required the initial steps of resuscitation only. Her one-minute Apgar score was 6- and her five-minute score was 8. Her umbilical atrial cord pH was 7.22, bicarbonate 16, and base deficit 8.3.

**Figure 1:**
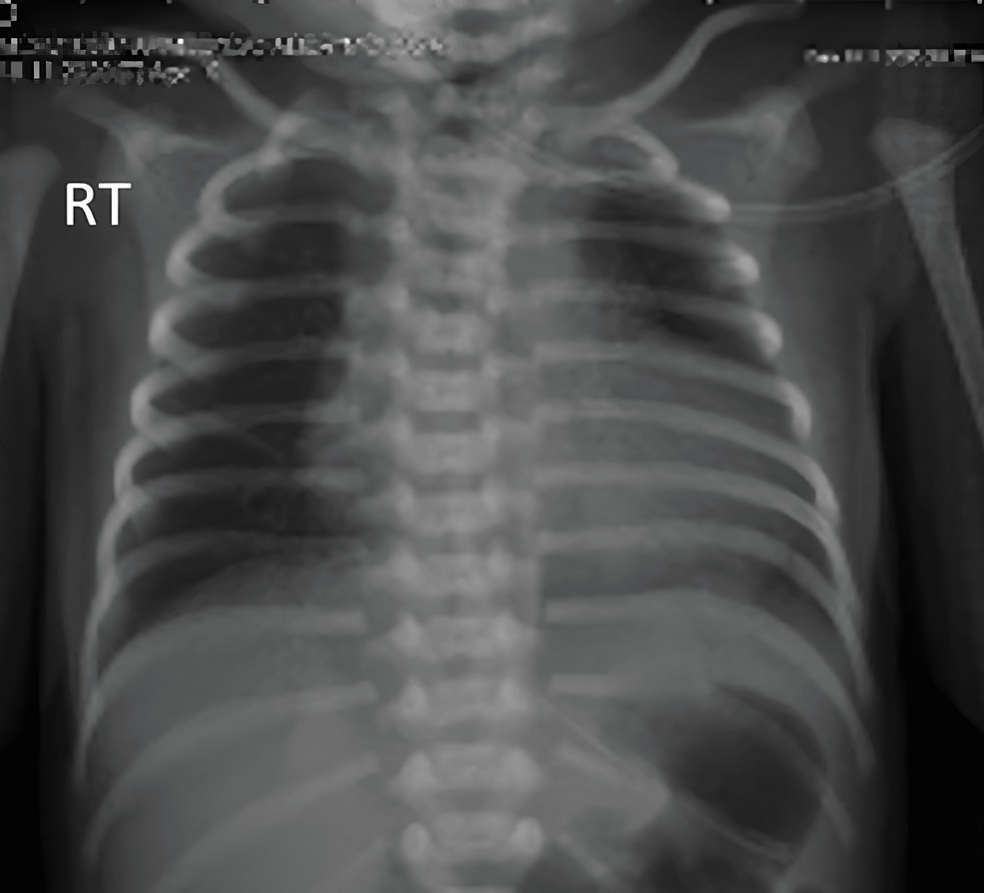
Chest X-ray during the first few hours of life showed mild increased interstitial markings in both lungs

She was admitted to the neonatal intensive care unit (NICU) due to respiratory distress and central hypotonia, requiring a nasal. Her chest X-ray showed mild increased interstitial markings in both lungs, which was suggestive of transit tachypnea of the newborn (Figure 1). Echocardiography showed a normal structural heart with no evidence of pulmonary hypertension. However, her blood culture showed no growth. Her prothrombin time was 12.4 seconds, and her partial thromboplastin time was 33 seconds, which was normal. Her newborn metabolic screen was reported as negative.

**Figure 2:**
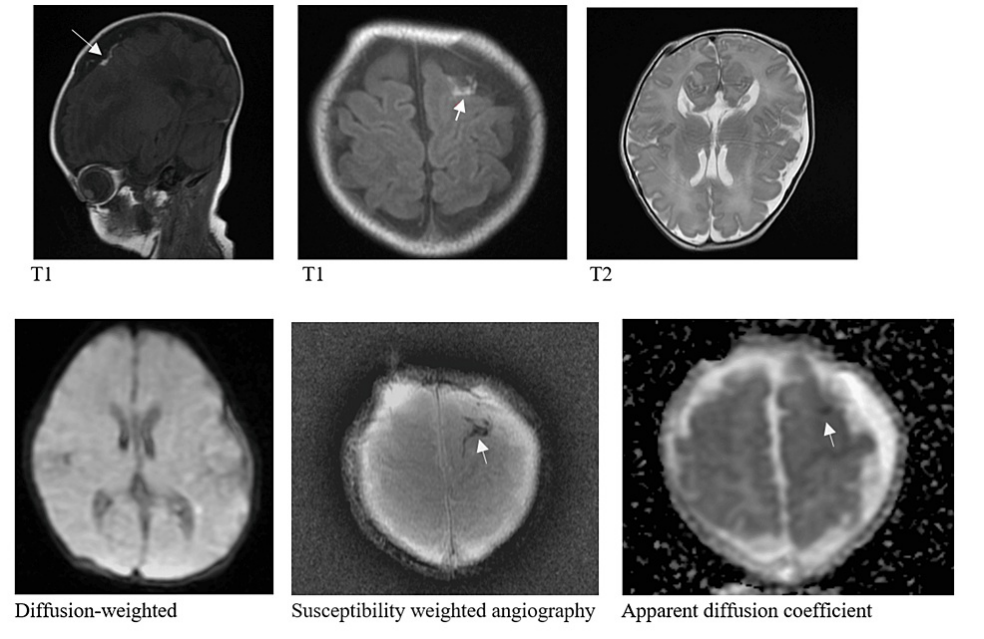
Brain magnetic resonance images at six days of life showed a small, left-sided subdural hemorrhage (arrows)

She was treated empirically with intravenous antibiotics for five days. Initially, she was started on intravenous fluid and when her respiratory distress resolved, she had an orogastric tube (OGT) for feeding because of severe hypotonia, which led to poor sucking and swallowing. Brain magnetic resonance was performed on day six of life, which was reported as normal, except for a small convexal subdural hemorrhage in the left precentral sulcus (Figure 2). As all her basic investigations were unremarkable, and this type of subdural hemorrhage is common and benign [6], a whole exome sequencing (WES) analysis was conducted when she was 22 days old at PerkinElmer Genomics. Two variants were identified: a nonsense variant c.966G>A (p.Trp322Ter) in CHD1 and a missense variant c.1723T>G (p.Ser575Ala) in ASH1L (absent small and homeotic disks protein 1 homolog like histone lysine methyltransferase).

She was discharged from the NICU on nasal cannula and OGT feeding but was later readmitted due to aspiration pneumonia. The patient was dependent on a nasal cannula for 30 days and required OGT feeding for 45 days. At present, she has been able to feed orally without any difficulties and has not experienced any respiratory issues. The patient underwent physiotherapy for 18 months, which we believe aided in her ability to walk. The patient is presently three years old, and Table 1 provides information on her baseline demographic characteristics as well as the developmental quotient (DQ) for each developmental domain at three years of age.

**Table 1:** Patient’s characteristics and developmental quotient (DQ) at three years of age

Characteristics	Value/Remarks
Weight	16 kg (falls at the 90^th^ percentile of the Saudi growth chart)
Height	87 cm (falls at the 10^th^ percentile of the Saudi growth chart)
Head Circumference	48 cm (falls between the 25^th^ percentile and 50^th^ percentile of the Saudi growth chart)
Depressed Midface	Present
Almond Shaped Eyes	
Flaring of Eyebrows	
Pointed Chin	
Hypotonia	
Autistic Features	
Seizure	Febrile convulsion, normal electroencephalography
DQ of Social/Emotional Domain	33
DQ of Language/Communication Domain	16.7
DQ of Cognitive Domain	16.7
DQ of Movement/Physical Development Domain	66
Speech Apraxia	No expressive language
Stereotypies	Not present
Down-Slanting Palpebral Fissures	
Fetal Fingers	
Skin Abnormalities	
Allergic Shiners	
Immune Abnormalities	
Skeletal Dysplasia	

The patient's parents are third-degree cousins from Al-Ahsa Governorate in the Eastern Province of Saudi Arabia. At the time of conception of their daughter, the mother was 44 and the father was 50 years old. They also have three older daughters and four older sons who have no neurodevelopmental impairment. They were offered a WES, but they refused it, as they felt it wouldn't be of much help in their daughter's care or prognosis. Furthermore, they did not consent to the use of their daughter's images for publication.

## Discussion

We are reporting on a unique case from Saudi Arabia, which exhibits a phenotype and genotype that are consistent with PBS. What makes this case stand out is that both CHD1 and ASH1L variants coexist. The CHD1 variant is novel. The case shows developmental dissociation, which could be attributed to the ASH1L variant. This particular case is of a female patient, adding to the evidence of female-skewing in PBS. However, the mechanism behind this phenomenon is yet to be explored.

In Table 1, we compared and contrasted the phenotypic features of our patient with those of PBS. The following features were common in our patient and other reported cases of PBS: autistic features, global developmental delay, hypotonia, depressed midface, almond-shaped eyes, and flaring eyebrows. The unavailability of the daughter's images due to the parent's refusal to give consent might have a significant impact on comprehension of the craniofacial dysmorphism related to PBS. While growth retardation is a feature of PBS [1], our patient's weight at three years of age was 16 kilograms, which falls at the 90th percentile of the Saudi growth chart [7]. This difference could be due to the variability within PBS [1,2], or it could be due to the parent's insist on feeding their daughter a high-caloric milk formula. Our patient and five of the six reported cases of PBS are female, and the reason for this female skewing is still unknown [1]. However, it has been hypothesized that male individuals may exhibit a lower tolerance to CHD1 variants [1].

Our patient's developmental delay is severe, as her developmental quotient (DQ) is less than 70 in every developmental domain [8]. A developmental dissociation is defined as a significant difference between the developmental rates of two or more domains of development, with one domain being significantly more delayed [8]. Based on this definition, our patient has a developmental dissociation as her DQ differs between the four developmental domains, with the language and cognitive domains being more delayed than the other three domains (as shown in Table 1). It is worth noting that developmental dissociation was not reported in the previous cases of PBS.

Despite our patient had fetal distress and meconium-stained amniotic fluid, there was no evidence of hypoxia ischemic encephalopathy, as she required only initial steps of resuscitation, her five minutes was 8, and her umbilical arterial cord blood gas was normal [9]. Our patient's MRI brain showed a small convexal subdural hemorrhage in the left precentral sulcus (Figure 2). This type of subdural hemorrhage is common with a prevalence of 8%-25 % and is not associated with neurodevelopmental impairment [6]. Therefore, we believe that the fetal distress and subdural hemorrhage were not contributing factors to the severe hypotonia, severe developmental delay, and developmental association that were observed in our patient.

The oldest reported case of PBS is a 17-year-old male who has severe developmental delay and intellectual disability [2]. Our current case, which is now three years old, presents a challenge in determining its long-term prognosis. Therefore, it is crucial to closely monitor the condition and evaluate potential long-term outcomes. Both the parents and we have considered long-term physiotherapy as a possible intervention to address severe hypotonia. However, this requires further investigation and analysis to determine the most effective course of treatment for the patient.

The patient we are discussing is the first known case in the world to have a CHD1 variant of c.966G>A (p.Trp322Ter) [10]. In contrast to the previously reported six cases of PBS that had a missense variant, our patient is the first known case of PBS with a nonsense variant [1,2]. Missense variants in the CHD1 gene are generally poorly tolerated and may lead to disruptions in protein structure and function [1,11]. However, mice with the deletion of a single CHD1 allele maintain normal phenotypes, which implies that further research is necessary to elucidate the consequences of loss of function variants [1,11].

The CHD1 variant in PBS appears to be de novo, as was the case in three previously evaluated patients [1,2]. According to existing literature, PBS is inherited in an autosomal dominant pattern [2,11]. Our patient's parents are third-degree cousins, indicating remote consanguinity [12]. However, it is important to note that consanguinity does not significantly impact autosomal dominant disorders [12]. Due to the lack of WES results for the parents, we are unable to confirm whether the CHD1 variant in our patient is de novo and what mode of inheritance is implicated in our patient.

Our patient has a heterozygous missense variant in ASH1L c.1723T>G (p.Ser575Ala), which may be associated with intellectual disability, autosomal dominant 52 (OMIM 617796). If this assumption is proven correct, the ASH1L c.1723T>G (p.Ser575Ala) variant could be the reason for the developmental dissociation in our patient. It is worth noting that the ASH1L variants were not reported in the previous cases of PBS [1,2]. It is imperative to investigate to determine whether the coexistence of CHD1 and ASH1L variants is contributing to the phenotypic expressions observed in our patient. Moreover, ASD is more likely to occur in children with parents over 40 years of age [13,14]. As our parents were both over 40 years old at the time of conception of their daughter, this may have added to the risk of ASD in addition to the CHD1 variant. However, ASD is a multifaceted condition that arises from a combination of genetic and environmental factors, as well as gene-environment interactions [15]. The intricate nature of the disorder necessitates a thorough investigation of the complex interplay between these elements [15].

The genetic variation landscape is vast, with the majority of it having a neutral effect on the phenotype [16-18]. However, a small fraction of the variations can be harmful [17,18]. To avoid incorrect clinical decisions based on common variants, it is imperative to have a strategy in place to interpret the clinical significance of rare or novel variants appropriately [16-18].

To this end, in 2015, the American College of Medical Genetics and the Association for Molecular Pathology (ACMG) issued a consensus guideline that combined computational, functional, population, and clinical data as criteria to stratify the strength of evidence and to determine the pathogenic status [18]. The ACMG endorsed the nonsense variant as very strong evidence of pathogenicity, as it can often be assumed to disrupt gene function [18]. It also supported that the de novo variant is considered strong support for pathogenicity [18]. Additionally, the ACMG suggested that a prior observation of the very rare variant in multiple unrelated patients with the same phenotype may be used as a moderate level of evidence of pathogenicity [18].

Our patient’s single nucleotide CHD1 variant satisfies the ACMG criteria except that the de novo status is unknown due to the lack of WES results for the parents [18]. Therefore, we consider our patient’s CHD1 variant to be pathogenic. Our patient's single nucleotide variant ASH1L variant is rare, as it is found in about 0.04% of the general population [19], which is < 0.5% [17]. This ASH1L variant is unprecedented as a pathological variant. Hence, according to ACMG, our patient's ASH1L variant is of uncertain significance [18].

It is important to analyze very large numbers of genome sequences to identify medically important rare genetic variants [17,18]. Functional studies can be a powerful tool in support of pathogenicity [17,18]. Estimating how much of the genome is functionally important is not straightforward. The traditional way is to carry out cross-species comparisons to identify how much of the genome is subject to purifying selection to conserve functionally important sequences [17,18]. Population-based human genome sequencing also offers insights into evolutionarily recent functional constraints [17,18].

## Conclusions

We present the first reported case of PBS in Saudi Arabia that demonstrates a genotype and phenotype consistent with this condition. In this case, a notable feature is the developmental dissociation observed along with the coexistence of CHD1 and ASH1L variants. The CHD1 variant observed in this case is a novel finding. The developmental dissociation observed in this patient may be attributed to the ASH1L variant. Furthermore, the fact that the patient is female supports the female-skewing phenomenon observed in PBS, although the underlying cause of this phenomenon necessitates further investigation. This report highlights the importance of identifying and characterizing rare genetic disorders such as PBS. Understanding the genetic basis of these disorders can lead to improved diagnosis, treatment, and management strategies. Continued research on the genetic and molecular mechanisms underlying PBS and related disorders is crucial for advancing our knowledge and developing effective therapies.
